# From the Identification to the Dissection of the Physiological Role of the Mitochondrial Calcium Uniporter: An Ongoing Story

**DOI:** 10.3390/biom11060786

**Published:** 2021-05-23

**Authors:** Giorgia Pallafacchina, Sofia Zanin, Rosario Rizzuto

**Affiliations:** 1 Department of Biomedical Sciences, University of Padua, 35131 Padua, Italy; 2 Neuroscience Institute, Italian National Research Council (CNR), 35131 Padua, Italy; 3 Department of Immunology, Infectiology and Haematology, Institut Necker-Enfants Malades (INEM), INSERM U1151-CNRS UMR 8253, 75015 Paris, France; sofia.zanin@inserm.fr

**Keywords:** MCU, mitochondrial Ca^2+^ uniporter, Ca^2+^ signaling, mitochondrial metabolism, skeletal muscle mitochondria

## Abstract

The notion of mitochondria being involved in the decoding and shaping of intracellular Ca^2+^ signals has been circulating since the end of the 19th century. Despite that, the molecular identity of the channel that mediates Ca^2+^ ion transport into mitochondria remained elusive for several years. Only in the last decade, the genes and pathways responsible for the mitochondrial uptake of Ca^2+^ began to be cloned and characterized. The gene coding for the pore-forming unit of the mitochondrial channel was discovered exactly 10 years ago, and its product was called mitochondrial Ca^2+^ uniporter or MCU. Before that, only one of its regulators, the mitochondria Ca^2+^ uptake regulator 1, MICU1, has been described in 2010. However, in the following years, the scientific interest in mitochondrial Ca^2+^ signaling regulation and physiological role has increased. This shortly led to the identification of many of its components, to the description of their 3D structure, and the characterization of the uniporter contribution to tissue physiology and pathology. In this review, we will summarize the most relevant achievements in the history of mitochondrial Ca^2+^ studies, presenting a chronological overview of the most relevant and landmarking discoveries. Finally, we will explore the impact of mitochondrial Ca^2+^ signaling in the context of muscle physiology, highlighting the recent advances in understanding the role of the MCU complex in the control of muscle trophism and metabolism.

## 1. Introduction

Every cell type, in every tissue and at any evolutionary level, can communicate with the surrounding environment and with neighboring cells. Both intercellular and extracellular communication play fundamental roles in shaping cell behavior and driving cell fate decisions. Cell-to-cell and environmental signals are normally conveyed by distinct extracellular mediators (hydrophilic or hydrophobic compounds, mechanical, ionic, cell–cell interactions, etc.) that are normally perceived by cells through surface receptors. These receptors convey them into a limited number of intracellular molecules, which are referred to as ‘second messengers’, which, in turn, forward the message to intracellular effectors finally activating the ultimate cellular responses. Despite the plethora of different signals and stimuli that cells may receive, only a few molecules to date have been described as second messengers of intracellular communication. Among them, the most common and, definitively, the most extensively studied is Ca^2+^.

Ca^2+^ ions participate in the decoding of a vast range of stimuli and the variety of cellular components involved in the Ca^2+^ signal transduction is extremely wide, including basically all kinds of components, organelles, and molecules [1,2,3]. The research studies on Ca^2+^ second messenger started more than one hundred years ago, with the initial recognition of the role of Ca^2+^ in muscle cell contraction [4]. Since then, the understanding of Ca^2+^ signaling regulation and dynamics has progressively increased leading to the definition of the concept of Ca^2+^ compartmentalization and to the demonstration of the existence of microdomains of local high Ca^2+^ concentration [5], which are crucial for the fine-tuning and correct triggering of the Ca^2+^-dependent cellular effects [1].

Mitochondria play a fundamental and multifaceted role in the orchestration of cellular Ca^2+^ signals. Indeed, mitochondria are not a store of rapidly releasable Ca^2+^ (such as the ER), but rather they efficiently accumulate Ca^2+^ upon Ca^2+^ entry from the extracellular space or upon release from ER Ca^2+^ stores [6]. Upon cytosolic Ca^2+^ elevation, the entry of Ca^2+^ into mitochondria exerts a central function in the modulation of cell metabolism. Mitochondria host the enzymes and complexes of the TCA cycle, fatty acid oxidation (FAO), and oxidative phosphorylation (OXPHOS) thus representing the site of the major metabolic pathways and enzymes for cell energy supply, which deserved them the name of ‘cellular powerhouses’. Interestingly, Ca^2+^ entry and oxidative activity are two strictly intertwined aspects of mitochondrial physiology. The increase of the mitochondrial matrix Ca^2+^ level stimulates both Ca^2+^-sensitive dehydrogenases [7,8,9] and respiratory chain complexes [10,11] resident in the organelles, fueling the TCA cycle activity as well as aerobic respiration and thus boosting the overall oxidative metabolism. This makes mitochondria the central hubs for the rapid and effective adaptation of cell metabolism to the changes in energy requirements that are typically decoded as variations of intracellular Ca^2+^ concentration.

In addition, mitochondria also actively participate in the tuning of global Ca^2+^ signals thanks to their ability to take up Ca^2+^ during intracellular Ca^2+^ elevation with a net result of buffering the cytosolic cation concentration thus modulating the overall cellular Ca^2+^ response. This buffering capacity is due to two crucial characteristics of the mitochondria: (i) their strategic position in close contacts to the Ca^2+^ release channels of the ER store [6] and the plasma membrane in immune cells [12] (ii) the presence on their inner membrane of highly selective and efficient machinery for taking up Ca^2+^, the MCU complex.

Finally, the large buffering capacity of mitochondria can protect cells from Ca^2+^ overload. Indeed, an excessive accumulation of the cation in the mitochondrial matrix triggers the permeability transition pore (PTP) opening, the release of pro-apoptotic factors, and finally, induction of programmed cell death [13]. Given the strong association between mitochondrial Ca^2+^ overload and apoptosis induction, the maintenance of mitochondrial Ca^2+^ homeostasis is thus a crucial aspect for ensuring cell survival [14,15].

**Figure 1:**
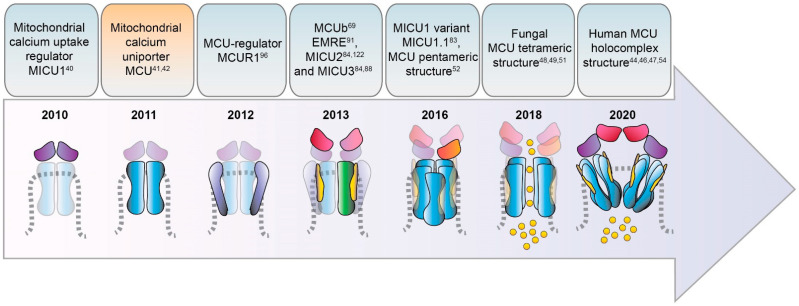
Timeline of the identification of MCU complex components. The most relevant findings on the structure and composition of the MCU complex discovery are summarized and chronologically presented along a timeline covering the last 10 years. Schematic cartoons show the different components of the MCU complex according to the date of their discovery along the timeline.

Given the extreme relevance of mitochondria Ca^2+^ signaling for cell physiology, the unveiling of the molecular factors mediating mitochondrial Ca^2+^ entry and the mechanism(s) of their regulation has been one of the scientific challenges of recent years. In this review, we aim to summarize some of the milestone achievements in the history of mitochondrial Ca^2+^ research with a particular focus on the recent findings of the mitochondrial Ca^2+^ uniporter and its role in organ physiology. We will briefly describe the early studies leading to the demonstration of the Ca^2+^ accumulation capacity of mitochondria, then we will go through the historical chronicle of the discoveries of the mitochondrial Ca^2+^ uniporter genes and multiple regulators (Figure 1) and we will conclude with an excursus on the physiological relevance of mitochondrial Ca^2+^ uptake in the context of skeletal muscle tissue.

## 2. Timeline of MCU Identification

It is suggestive to recall that the notion of Ca^2+^ ions being relevant to organ physiology dates back to more than one century ago when the first report on the physiological action of Ca^2+^ ions appeared in 1883 [4]. At that time, Ringer described the effects of Ca^2+^ addition to isolated frog hearts and demonstrated that the supplementation of Ca^2+^ in the perfusion solution actively induces and sustains the contraction of the organ ex vivo [4]. This seminal observation revealed that Ca^2+^ is a fundamental messenger within cells, a concept that then extended to virtually every cell type and physiological and pathological process, giving rise to a broad field of study commonly referred to as the field of intracellular Ca^2+^ signaling. The intrinsic ability of contracting myocytes to operate ex vivo and to rapidly and effectively respond to environmental condition changes made them the ideal experimental system for the investigation of the role of Ca^2+^ in organ and cell physiology and was extensively exploited by researchers in the following years.

The original concept of the existence of intracellular compartments acting as Ca^2+^ stores to accumulate the cation required to sustain muscle contraction has been later postulated and demonstrated in 1947 by Heilbrunn [16]. However, although surprising, the identification of the sarco/endoplasmic reticulum (SR/ER) as the principal cellular Ca^2+^ store came only 20 years later. It was in the 1960′s, with the identification of Ca^2+^ pumping machinery on intracellular membranes (in particular the calcium pump of the sarcoplasmic reticulum, better known as SERCA) by three independent scientists [17,18,19,20] and the advent of new methodologies for the measurement of intracellular Ca^2+^ concentration [21] that the ER and its specialized counterpart in muscle cells (the SR) were recognized the main cellular reservoir of Ca^2+^.

Before that, the pioneering work of Slater & Cleland on cardiac myocyte preparations from rat hearts firstly described some subcellular compartments, called "sarcosomes" at that time, as the entities actively accumulating Ca^2+^ [22]. Interestingly, these “sarcosomes” did not consist of ER but, instead, they corresponded to isolated mitochondria, to which the addition of Ca^2+^ caused the block of their oxidative phosphorylation activity. Thus, Ca^2+^ ions behaved as mitochondria uncouplers. Despite that, Ca^2+^ appeared to exert a peculiar inhibitory action on mitochondrial OXPHOS activity, which differed from the other irreversible uncouplers known at that period (dinitrophenol, dicoumarol, rotenone, antimycin A, azide, or cyanide), due to the reversibility of its action [23]. This assigned to Ca^2+^ ions a functional role in mitochondria activity.

After that, a series of land-marking works in the early 60′s experimentally revealed that energized mitochondria can actively take up Ca^2+^ [24,25,26]. Interestingly, these results anticipated the clear demonstration of a driving force for Ca^2+^ accumulation in mitochondria, i.e., the chemiosmotic theory, postulated and validated by Peter Mitchell [27]. This theory is based on the following concepts: (i) the activity of the respiratory chain complexes is linked to the extrusion of protons from the matrix to the intermembrane space (IMS) across the inner membrane of mitochondria (IMM). (ii) The accumulation of protons in the IMS generates a difference in the charges across the IMM of around −150 ÷ −180 mV (negative inside) establishing the so-called mitochondrial membrane potential (ΔΨ_m_). (iii) This steep ΔΨ_m_ represents the main driving force for the proton gradient-sustained synthesis of ATP and Ca^2+^ cation entry into the matrix.

Shortly after, at the University of Bristol, Denton and his group made important discoveries on the Ca^2+^-dependent modulation of three critical oxidative enzymes resident in mitochondria [7,8,9]: pyruvate dehydrogenase phosphatase (the enzyme that dephosphorylates and relieves pyruvate dehydrogenase (PDH) activity allowing the conversion of NAD^+^, coenzyme A (CoA) and pyruvate into NADH, CO_2_, and acetyl-CoA, providing substrates to the citric acid (TCA) cycle and cellular respiration), NAD-isocitrate dehydrogenase and oxoglutarate dehydrogenase. The fact that these mitochondrial rate-limiting enzymes are under the control of Ca^2+^ definitively sets the cation at the center of the cell oxidative metabolism. Moreover, the evidence that mitochondria can take up Ca^2+^ in response to elevation of cytosolic Ca^2+^ levels, as shown in insulin-treated epididymal adipose tissue [28], established an active role of mitochondrial Ca^2+^ entry in shaping cell oxidative metabolism to match the increased cellular energy demands thus tailoring the metabolic outcomes according to the different environmental cues.

Despite all these conceptual advancements, two major issues were destined to puzzle the scientific community for decades. On one end, there is the apparent paradox between the physiological concentration of cytosolic Ca^2+^, which was estimated in the submicromolar range [29,30,31], and the low affinity of the mitochondrial Ca^2+^ uptake, whose half-maximal rate (Km) was measured in the order of several µM (reviewed in [32]). On the other end, the fundamental question about the molecular identity of the IMM apparatus responsible for the entry of Ca^2+^ into mitochondria was still without an answer.

It took around 30 and 50 years, respectively, to find solutions to those two enigmas. The answer to the former came with the advent of innovative and sophisticated technologies allowing the assessment of Ca^2+^ distribution at the sub-cellular and sub-organellar levels, including mitochondria. Indeed, thanks to the development of Ca^2+^-sensitive genetic probes and recombinant fluorescent proteins targeted to specific intracellular microdomains [33,34], it was possible to measure variations of Ca^2+^ concentration in defined and limited areas of the cell (such as the cytosolic face of plasmalemma [35] or the surface of outer mitochondrial membrane [36], or the Golgi cisternae [37]) as well as the relative positioning of the intracellular organelles. These studies were pivotal in the field of cell biology for two main reasons: i) they allowed the observation of the intrinsic heterogeneity of cellular Ca^2+^ distribution, definitively demonstrating that large variations in Ca^2+^ concentration are highly regionalized within the cell cytoplasm and allowing the direct measure of Ca^2+^ levels in the mitochondria matrix as well as in the ER lumen; ii) they pinpointed the fact that organelles, including ER and mitochondria, are in close contact with each other through macromolecular structures involving proteins from both the compartments. In the case of ER-mitochondrial contacts, these structures are biochemically isolated as mitochondria-associated membranes (MAMs) and are formed by membrane channels, as the IP_3_R and VDAC, respectively, and adaptor proteins of both organelles, such as Grp75, mitofusins, PACS [38]. Upon cell stimulation, the massive release of Ca^2+^ through the ER membrane clusters of IP_3_Rs generates microdomains of high Ca^2+^ concentration right at the mouth of the channel pores, exactly where mitochondria are located. This allows mitochondria to perceive a local cation concentration sufficient to meet the low affinity of the mitochondrial Ca^2+^ uptake machinery [36,39]. Thus, their strategic position in proximity of ER Ca^2+^ release channels and their ability to take up Ca^2+^ with high conductance make mitochondria the ideal operator for cushioning the sudden Ca^2+^ rise in the cytosol of stimulated cells, thus behaving as an instrumental Ca^2+^ buffer [6]. The fact that Ca^2+^ entry into mitochondria stimulates the TCA cycle, respiration, and ATP production then places mitochondrial Ca^2+^ uptake as a key element for the prompt modulation of cell metabolism to rapidly and efficiently adapt to a variety of environmental cues and energy demands.

Another fundamental advancement in mitochondrial signaling occurred when scientists found the answer to the second big question, i.e., the molecular identity of the mitochondrial Ca^2+^ uniporter (MCU) machinery. The chronicle of MCU discoveries actually started in 2010, with the identification of the first gene required for the uptake of Ca^2+^ by mitochondria, *CBARA1*, coding for the mitochondrial Ca^2+^ uptake 1 protein (MICU1) [40], then followed by the identification of the mitochondrial channel and the elucidation of its interactors, as described below. The search of the other mitochondrial Ca^2+^ channel components has been proceeding expansively in the last decade (see next paragraph for a detailed timeline) and it is presently still actively ongoing. The discoveries of many different groups worldwide have been indeed instrumental to provide cell biologists with new knowledge on the functional role of mitochondrial Ca^2+^ and with new tools for the genetic and molecular intervention on global Ca^2+^ signaling and cell energetics.

## 3. Discovery and Characterization of the MCU Complex Components

### 3.1. MCU

The chronicle of MCU discovery starts with two pioneering studies published in 2011 [41,42] (Figure 1) that finally identified and cloned the long-sought MCU pore-forming unit gene, *CCDC109A*. MCU is a highly conserved 40 kDa protein ubiquitously expressed in plants, metazoans, protozoans, and fungi but not present in yeast [43]. This pore-forming unit oligomerizes to form the active channel within the inner mitochondrial membrane (IMM) and it directly interacts with the channel regulator MICU1, which was identified one year earlier [40]. These reports clearly demonstrated that the transient downregulation of MCU inhibits the mitochondrial accumulation of Ca^2+^ that follows the IP_3_-generating agonist in stimulated cells. Of note, the blunted mitochondrial Ca^2+^ uptake response occurs without changes in the mitochondria morphology or membrane potential of the MCU-silenced cells [41]. On the contrary, MCU overexpression enhances agonist-induced mitochondrial Ca^2+^ uptake in mammalian cells. In addition, in vitro experiments, in which recombinant MCU proteins were inserted in a planar lipid bilayer, showed that this pore-subunit alone is sufficient to form the channel. Indeed, in this setting, the MCU electrophysiological activity is completely abolished by the addition of the known inhibitor Ruthenium Red, firmly pointing at MCU as the genuine core component of the mitochondrial Ca^2+^ machinery.

Sequence and topology analyses revealed that both the *N*- and *C*-termini of MCU are located in the mitochondrial matrix and that MCU is endowed with two transmembrane domains, linked by a short highly conserved acidic loop exposed in the intermembrane space (IMS) which contains the so-called acidic “DIME” motif. The acidic residues present in this stretch (in particular E257, D261, E264) are critical for the Ca^2+^ transport since, if substituted with uncharged residues, MCU mutants failed to rescue the mitochondrial Ca^2+^ uptake in MCU-silenced cells [41,42]. However, the definitive description of MCU 3D structure had to wait till the very last years, when cryo-EM and X-ray diffraction analyses finally allowed the resolution of full-length MCU structure [44,45,46,47,48,49,50,51]. These studies coherently confirmed that purified MCU from different sources (fungi and metazoan) arranges in a tetramer, confuting previous assumptions on a putative pentameric MCU architecture [52]. Notably, the cryo-EM data also unveiled the exact position of the MCU channel selectivity filter, in which the DIME motif is fundamental for the coordination of Ca^2+^ ions and which was definitively shown to reside at the beginning of the second transmembrane α-helix [50] and not in the linker region between the two transmembrane helices, as previously suggested [52].

**Figure 2:**
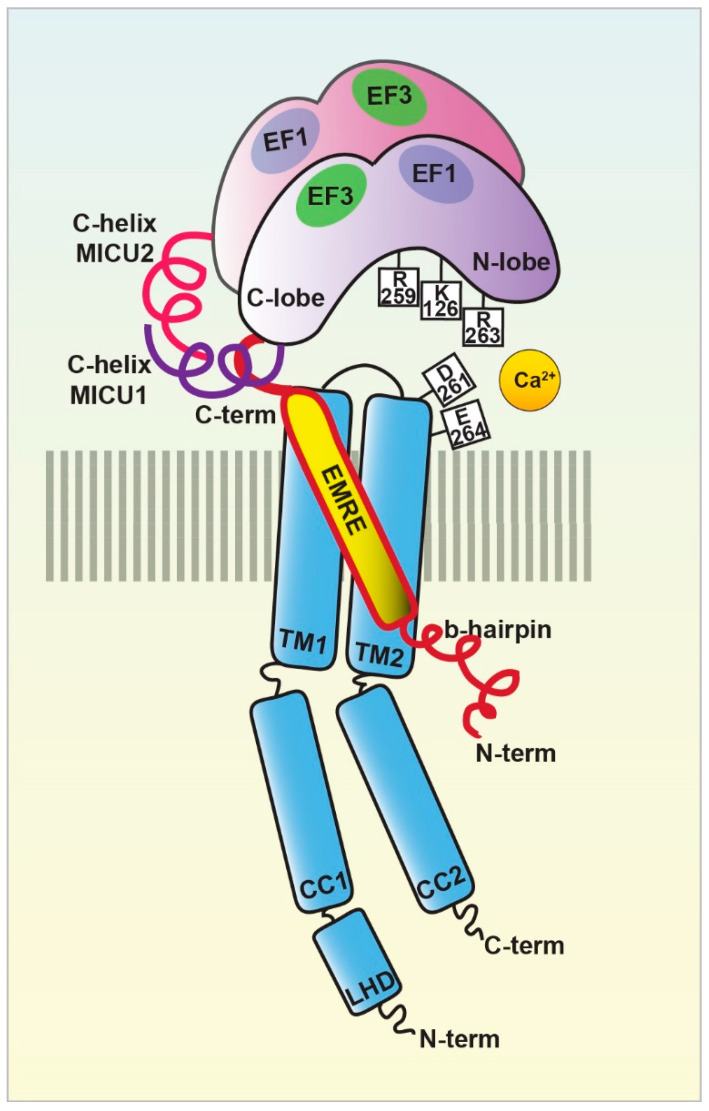
The MCU holocomplex structure. Schematic representation of the MCU holocomplex (uniplex) components and their relevant domains: the pore-forming subunit MCU (light blue) with the two transmembrane (TM) and coiled-coil (CC) domains and the linker helix domain (LHD); the essential mitochondrial Ca^2+^ uniporter regulator EMRE (yellow); the mitochondrial Ca^2+^ uptake proteins MICU1 (violet) and MICU2 (purple), with the EF-hands relevant for the MICU dimer interaction highlighted. The critical residues of the MCU DIME motif forming the Ca^2+^ selectivity filter are indicated, together with the MICU1 residues of the K-R ring coordinating the MCU acidic region.

More recently, the structure of the human MCU together with its auxiliary component EMRE was obtained [53]. Each human MCU arranges in tetramers and each subunit complexes with one EMRE peptide. Differently from the three described for fungal MCU, human MCU appears organized in four domains, which are: (i) the *N*-terminal domain (NTD), (ii) the linker helix domain (LHD) —absent in fungi—, (iii) the coiled-coil domain (CCD), and iv) the transmembrane domain (TMD) (Figure 2).

**Figure 3:**
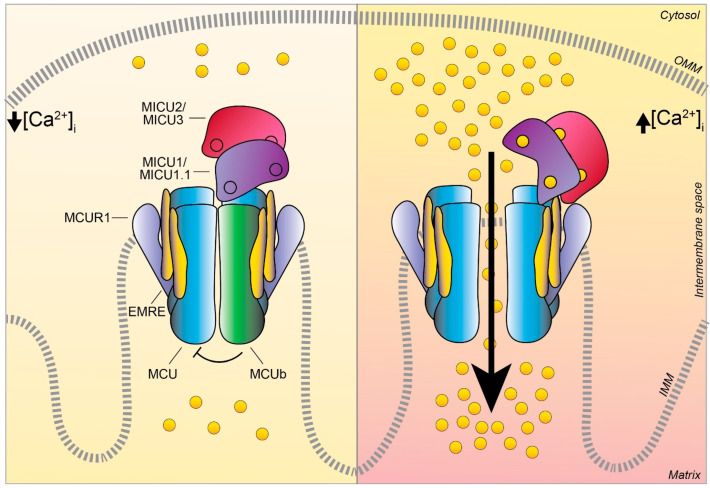
The MCU complex activity at low and high intracellular Ca^2+^ concentration. Schematic representation of the proteins involved in the MCU complex-mediated mitochondrial Ca^2+^ uptake: the pore-forming subunits MCU (light blue) and MCUb (green), the essential mitochondrial Ca^2+^ uniporter regulator EMRE (yellow), the mitochondrial Ca^2+^ uptake proteins MICU1 / MICU1.1 (violet), MICU2/ MICU3 (purple), and the MCU regulator 1 MCUR1 (light violet). The EF-hand Ca^2+^ binding domains of MICU proteins are indicated as little circles. At low intracellular Ca^2+^ concentration, the cation does not permeate through the MCU channel since the heterodimers formed by MICU1/MICU1.1–MICU2/MICU3 block the channel pore, thus preventing Ca^2+^ flux in resting conditions. Differently, at high intracellular Ca^2+^ concentration, MICU proteins undergo conformational changes relieving the inhibition on MCU and positively regulating channel activity, leading to an efficient mitochondrial Ca^2+^ uptake. OMM, outer mitochondrial membrane; IMM, inner mitochondrial membrane.

Moreover, in the very last year, further insight into MCU channel modulation and function has been gained thanks to the achievement of the human MCU-MICUs holocomplex structure in both the Ca^2+^-free and Ca^2+^-bound state by several independent reports. The gating mechanism by which MICU1 regulates the uniporter activity via the conformational change triggered by Ca^2+^ was finally unveiled [44]. Furthermore, the precise description of the molecular interactions between MCU-EMRE-MICU1-MICU2 in the human MCU supercomplex (MEMMS) has been also obtained [54] (Figure 2). Indeed, MEMMS appears as a 480 kDa integral unit where EMRE coordinates the matrix gate of the MCU channel and MICU proteins interact with the *C*-terminus of EMRE in the IMS thus enhancing Ca^2+^ influx through the MCU pore in high [Ca^2+^] conditions [54] (Figure 3). Finally, the distinct Ca^2+^-dependent assembly conformations of the beetle and human MCU holocomplexes with human MICUs have also been detailed [46,47]. In the presence of Ca^2+^, the multiprotein complex shows a two-fold symmetry and consists of two V-shaped MCU-EMRE tetrameric subcomplexes and two MICU1-MICU2 heterodimers that bridge the tops of the subcomplexes (Figure 1). In this setting, the assembly of the MICU1-MICU2 heterodimers to the MCU-EMRE subcomplexes is ensured by the interaction between MICU1 and EMRE [47] (Figure 2). Differently, in the absence of Ca^2+^, the holocomplex adopts alternative less stable conformations with both monomeric and dimeric forms of the MCU-EMRE tetramers, where the MICU1-MICU2 heterodimer block the channel entrance formed by MCU transmembrane domains [47] (Figure 2 and Figure 3).

A large body of experimental evidence on the MCU complex functional role has accumulated since the discovery of the MCU. The genetic manipulation of the MCU led to the generation of germline and tissue-specific transgenic models [55,56,57,58,59,60,61,62], which provided pivotal tools for understanding the pathophysiological implications of the mitochondrial Ca^2+^ signaling in vivo that would have otherwise remained unexplored.

In 2013, three independent groups showed that the *MCU* silencing/knockout or its overexpression affect survival in different in vivo models [55,63,64]. In *Trypanosoma brucei*, for instance, the downregulation or the conditional knockout of the uniporter augments the AMP/ATP ratio, thus affecting the parasite growth in vitro. On the contrary, when MCU is overexpressed, mitochondrial Ca^2+^ accumulation produces a high concentration of ROS and sensitizes trypanosomes to apoptotic stimuli [63]. In zebrafish (*Danio rerio)*, instead, the knockdown of MCU has shown crucial alterations not only during the early step of gastrulation, where the blastomere convergence and extension are altered, but also at later stages of development as the maturation of the notochord and anteroposterior axis formation were strongly impaired in morphant fish [64]. A series of recently published works and our unpublished observation in the zebrafish knockout model obtained with CRISPR/Cas9 gene ablation gave further insight into the role of MCU during development. For example, the ablation of the *MCU* gene in *Danio rerio* inhibits mitochondrial Ca^2+^ influx, reduces oxidative phosphorylation, and induces lipid accumulation, a phenotype that was also observed in the hepatic tissue of liver-specific KO mouse [65]. Notably, the inactivation of *MCU* has shown to be protective in neurons of Parkinson Disease zebrafish genetic model (namely in *pink^−/−^* fish), suggesting the crucial role of the MCU-dependent mitochondrial Ca^2+^ load on neuronal fitness [66].

As for mammals, the MCU^–/–^ mouse [55] develops normally and displays minor defects without signs of impaired cell survival. Under stress conditions, relatively mild metabolic alterations were observed, such as increased plasma lactate levels in line with impaired exercise performance. However, the same group soon after those findings, showed that embryos from MCU^−/−^ mice in a pure C57BL/6 background were not viable, displaying embryonic lethality at around E 11.5–E 13.5, thus suggesting a major involvement of mitochondrial Ca^2+^ uptake in organ metabolism and organism development that was compensated in a mixed genetic background [67].

Of note, some years later, it has been reported that the block of MCU-dependent Ca^2+^ uptake affects *Drosophila melanogaster* development. In particular, the inhibition of the uniporter activity has been shown to be detrimental for memory establishment during the papulation stage. Indeed, during the development of adult flies, alterations in the structural and functional neuronal substrates, crucial for memory formation, occur in the MCU deficient fly [58].

The genetic manipulation of MCU in *C. elegans* model also provided additional interesting notions on its role in organism physiology. Indeed, MCU^−/−^ *C. elegans* is viable and grossly normal, mirroring what was found in the first MCU^−/−^ mouse model described [55], even though it presents some defects in the epidermal wound repair mechanisms. More recently, the characterization of *C. elegans* deficient of a functional MCU [68] suggested that uniporter activity is essential for mitochondrial Ca^2+^ transfer during high-intensity stimulation of the worm pharynx muscle. However, a lot still remains to be explored on the role of MCU in this model and most of the knowledge on mitochondrial Ca^2+^ regulation of muscle physiology has been achieved using the mammalian mouse model where MCU expression was genetically targeted, which we will briefly review in the following paragraphs.

### 3.2. MCUb

In 2013, Raffaello and co-authors discovered that MCU is not the only pore-forming subunit of the mitochondrial Ca^2+^ uniporter, since an alternative MCU isoform exists, named MCUb, which crosses the IMM and associates to MCU to form the calcium channel [69] (Figure 1 and Figure 3). The MCUb protein is encoded by the MCU *CCDC109a* paralog gene *CCDC109b*. Interestingly, this gene is found in vertebrates, but it is not present in other organisms in which MCU is expressed, such as plants, Nematoda, and Arthropoda. Despite the high structural similarity with MCU, MCUb sequence presents two critical aminoacidic substitutions in the loop region and in the TM1 domain, which explains its inability to transport Ca^2+^. Indeed, MCUb acts as a negative regulator of MCU activity, drastically reducing mitochondrial Ca^2+^ currents in vitro in planar lipid bilayer experiments and also when overexpressed in mammalian cells [69]. On the contrary, in other organisms, such as trypanosomatid species, the ortholog of MCUb is capable to conduct the cation and its overexpression facilitates mitochondrial Ca^2+^ uptake [70].

**Figure 4:**
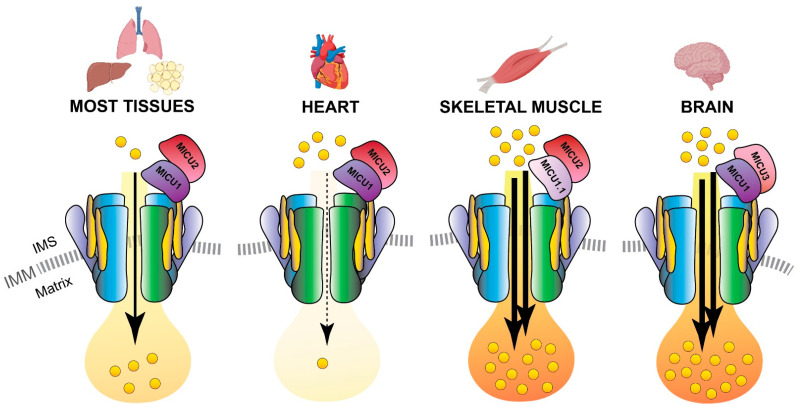
MCU holocomplex composition in different tissues. Schematic representation of the tissue-specific components of the MCU holo-complex. The presence of a relatively high MCUb:MCU ratio in the heart ensures a re-duced Ca^2+^ load in cardiomyocyte mitochondria. On the contrary, the expression of the MICU1.1 variant and MICU3 determines an elevated Ca^2+^ flux in the mitochondria of skeletal muscle fibers and neurons, respectively. IMS, inter-membrane space; IMM, inner mitochondrial membrane.

Interestingly, MCUb displays different expression levels in the different mammalian tissues, and the MCUb:MCU proportion appears also the distinctive feature ensuring the appropriate mitochondrial Ca^2+^ current to each cell type [69,71]. For instance, a high MCUb/MCU ratio (3:1) is typical of cells with low mitochondrial Ca^2+^ transients, such as adult cardiomyocytes (Figure 4). In fact, MCUb can be described as a protective gene in cardiac myocytes since i) its expression is transiently induced after ischemia-reperfusion injury and ii) transgenic mice overexpressing MCUb have a reduced mitochondrial Ca^2+^ uptake ability, thus preventing Ca^2+^ overload, which is sufficient to protect myocytes from ischemia-reperfusion injury and to decelerate their ongoing necrosis [72,73]. A low MCUb/MCU ratio (1:40) is instead a characteristic of tissues with an extremely high capacity of mitochondrial Ca^2+^ accumulation, such as skeletal muscle [69,74] (Figure 4).

These lines of evidence highlight the importance of MCU:MCUb proportion in the control of mitochondrial Ca^2+^ uniporter activity and further investigation will be of fundamental relevance for the understanding of its role in the pathophysiology of different tissues.

### 3.3. MICU1

MICU1 was actually the first component to be described as the regulator of the long-sought MCU channel and its identification even anticipated that of the pore-forming subunit MCU (Figure 1). Indeed, in 2010, an integrative strategy combining comparative physiology, evolutionary genomics, and organelle proteomics revealed the 54 kDa protein, encoded by the *CBARA* gene and residing in the IMS, to be the mitochondrial calcium uptake 1 (MICU1), which does not take part to the pore-forming domain of the channel, but it strongly regulates its activity in a Ca^2+^-dependent way [40,75]. Indeed, after the *N*-terminal mitochondrial targeting sequence, MICU1 shows two canonical EF-hand Ca^2+^ binding domains that confers the Ca^2+^-sensitivity (Figure 2 and Figure 3). In the last decade, several lines of evidence confirmed the initial hypothesis about the role of MICU1 as both MCU gatekeeper at low Ca^2+^ concentration and MCU positive regulator at high Ca^2+^ concentration, thus explaining the sigmoid cooperative effect of the MCU activation curve [75].

The downregulation of MICU1 was initially shown to abolish mitochondrial Ca^2+^ influx in intact and permeabilized HeLa cells [40]. Later studies also demonstrated that the absence of MICU1 also leads to an adaptive Ca^2+^ accumulation inside the mitochondria matrix, triggering excessive ROS production and the consequent higher sensitivity to apoptotic stress. In addition, the ability of MICU1 to sense cytosolic Ca^2+^ levels confers to MICU1 the capacity to set the threshold for the activation of mitochondrial Ca^2+^ uptake. Nevertheless, this occurs without altering the overall kinetics of the channel [75,76].

Two in vivo studies, performed in MICU1^-/-^ mouse models have strengthened this concept and gave additional insight into the physiological role of this MCU regulator. In more detail, the characterization of MICU1^-/-^ transgenic mice reveals that, despite partial postnatal mortality, the viable animals show marked ataxia and muscle weakness [77], a phenotype which is reminiscent of that of human patients bearing MICU1 genomic mutation [78]. Moreover, the MICU1^−/−^ mice display several biochemical defects, including an increase in resting mitochondrial Ca^2+^ levels, altered mitochondrial morphology, and a decreased ATP production [77]. Interestingly, the loss of MICU1 triggers a sustained pro-inflammatory response after partial hepatectomy and failure of liver regeneration in MICU1-deficient mice. In this scenario, the lack of MICU1 enhances mitochondrial permeability transition pore (PTP) opening in hepatocytes, thus leading to massive necrosis [79].

Interestingly, more recently, evidence of a new role of MICU1 in the regulation of crucial metabolic steps of cell metabolism has emerged. Indeed, MICU1 was shown to inhibit the mitochondrial transport of pyruvate and fatty acids and, interestingly, this MICU1 function appears independent of Ca^2+^ and MCU core-complex composition [80]. This study reveals a mechanism that controls the MCU-mediated Ca^2+^ flux machinery and that relies on TCA cycle substrate availability. According to this view, the MICU1 regulatory axis acts as a metabolic homeostatic circuit to protect cells from the risk of bioenergetic crisis and mitochondrial Ca^2+^ overload during periods of nutrient stress. Altogether these findings [77,79,80] highlight once more the crucial importance of the fine-tuning of mitochondrial Ca^2+^ uptake by Ca^2+^ and MICUs, especially in the context of promotion of cell survival under stress conditions.

In addition to that, new important functions of MICU1 in the control of mitochondrial cristae junctions have been revealed by the recent super-resolution structured illumination microscopy (SIM) and electron microscopy studies of Gottschalk and collaborators [81]. According to the authors, MICU1 appears to act as a Ca^2+^-dependent regulator of cristae junctions’ integrity and cytochrome c localization. Another intriguing feature of MICU1 regulatory function on the determination of uniporter cation selectivity has recently emerged [82]. MICU1 was revealed as the primary responsible for conferring and ensuring the stringent MCU selectivity for Ca^2+^ over Mn^2+^ since. Indeed, when present, MICU1 impedes Mn^2+^ ions to cross the MCU channel pore; on the contrary, in the absence of MICU1, both Ca^2+^ and Mn^2+^ cations can enter the mitochondrial matrix. This additional MICU1 checkpoint is of fundamental importance to guarantee cell survival of cells sensitive to Mn^2+^ such as neurons, thus setting MICU1 as a crucial safeguard against cellular toxicity due to manganese in neurodegenerative diseases [82].

### 3.4. MICU1.1

A variant of MICU1, named MICU1.1, has been discovered by our group as an alternative splicing product of the *MICU1* mRNA [83] (Figure 1). MICU1.1 is well-conserved among species and expressed almost exclusively in skeletal muscle tissue, where it is by far the most abundant MICU moiety (Figure 4), although limited but still appreciable levels are found also in the brain [83]. Similar to MICU1, MICU1.1 behaves as a positive regulator of MCU. Indeed, MICU1.1 expression increases mitochondrial Ca^2+^ uptake upon stimulation in HeLa cells and skeletal muscle in vivo, and this increase is even higher than that observed after expression of the conventional MICU1 isoform [83]. This behavior is explained by the fact that the muscular MICU1.1-MICU2 heterodimer (see next paragraph for a detailed description of MICU2), binds Ca^2+^ more efficiently than the canonical MICU1-MICU2 pair, thus activating the uniporter at lower Ca^2+^ concentrations [83]. This peculiar feature is of critical importance for the physiology of skeletal muscle tissue, and the presence of MICU1.1 is functional to ensure the Ca^2+^ uptake required for the matching of ATP production to the energy expenditure of muscle contractile activity [83]. These findings shed light on a novel modality by which MCU machinery can be modulated in skeletal muscle mitochondria and widen the scenario of the possible endogenous regulators of mitochondrial Ca^2+^ uptake, opening the path for future investigations on other tissue-specific isoforms and mechanisms controlling mitochondrial Ca^2+^ uptake.

### 3.5. MICU2

The first information about the existence of other genuine MCU regulators, in addition to MICU1, was provided by human genome sequencing studies a few years later [84] (Figure 1). Initially known as EF-hand domain-containing family member A1 (EFHA1), the mitochondrial calcium uptake protein 2 or MICU2, was found to be a paralog of MICU1 [84]. MICU2 resides in the IMS, contains two EF-hand Ca^2+^-binding domains, and interacts with both MICU1 and MCU [84] (Figure 2). The analysis of *MICU2* transcript expression shown that this regulator has a peculiar cell-type distribution: it is present at high levels in the intestine, prostate, and cardiac tissues [84]. Biochemical data evidenced that MICU2 stability is strictly dependent on the presence of MICU1: indeed, silencing of MICU1 leads to loss of also MICU2 protein, while MICU1 (or MCU) overexpression stabilizes both MICUs in mammalian cells [84]. Moreover, the recently reported human MICU1-MICU2 crystal structure [45,85] revealed interesting details on the architecture of the heterodimer and of MICU2 in both the Ca^2+^-bound and Ca^2+^-free condition. The MICU1-MICU2 interaction sites have been identified and correspond to Glu242 in MICU1 and Arg352 in MICU2 in the Ca^2+^-free state, while Phe383 in MICU1 and Glu196 in MICU2 contribute to the interaction of the two proteins in the Ca^2+^-bound state.

Although the functional role of MICU2 is still controversial, recent findings clarify some key aspects of uniporter modulation by this regulator. In mammalian cells, for example, it has been shown that MICU2 positively regulates MCU activation by controlling the cytosolic [Ca^2+^] threshold for the relief of MICU1-mediated inhibition of MCU. This function allows MICU2 to restrict the spatial Ca^2+^ crosstalk between inositol 1,4,5-trisphosphate receptor (InsP3R) and MCU channels [86].

Subsequently, the generation and characterization of the MICU2^−/−^ mouse model highlighted other important properties of this regulator [87]. MICU2 genetic ablation produces a decrease in the threshold for mitochondrial Ca^2+^ uptake due to loss of the gatekeeping activity and overall loss of MCU-dependent Ca^2+^ influx due to the destabilization of the entire uniporter complex. These findings lead to the conclusion that the amount of MCU and MICUs proteins is crucial to maintain the stability of the whole complex. Moreover, some phenotypic features of the MICU2^−/−^ mice are in common with models of cardiac pathologies, suggesting that MICU2 may act as a possible cardioprotective factor.

### 3.6. MICU3

MICU3, previously known as EFHA2, is another MICU1 paralog, originally identified by the same genetic sequence analysis that described MICU2 [84] (Figure 1). This finding added another level of complexity to the regulation of the MCU machinery. *MICU3* is an evolutionarily conserved gene since it is present in plants and in vertebrates, with a peculiar tissue-specific distribution: indeed, it is mainly expressed in the brain, much less expressed in skeletal muscle, and virtually absent in other tissues [88,89]. The *MICU3* gene encodes a 55 kDa protein that shares 34% and 47% protein sequence similarity with MICU1 and MICU2, respectively [84]. Like the other MICUs, also MICU3 has an *N*-terminal mitochondrial targeting sequence and binds Ca^2+^ thanks to the presence of EF-hand domains. The crystal structure of human MICU3 has been recently characterized in both Ca^2+^-free and Ca^2+^-bound conditions [90]. This crystallographic analysis revealed a MICU3 3D structure very similar to that of MICU2, in line with the role of these factors as MCU channel gatekeepers at low intracellular Ca^2+^ levels. Upon cytosolic Ca^2+^ increase, MICU heterodimers, including those containing MICU3, undergo a conformational change that releases the latch formed upon the uniporter mouth, thus allowing Ca^2+^ flux through the MCU pore [90] (Figure 3 and Figure 4).

Regarding its functional role, our group showed that MICU3 acts as a positive regulator of mitochondrial Ca^2+^ uptake through MICU1 [88]. Indeed, MICU3 forms heterodimers exclusively with MICU1, but not with MICU2, and the MICU1-MICU3 interaction leads to a significant increase of mitochondrial Ca^2+^ uptake, demonstrating the stimulatory action of MICU3 on uniporter activity [88]. Moreover, MICU3 downregulation blocks Ca^2+^ influx elicited by synaptic activity in primary cortical neurons, suggesting a specific role of this MCU regulator on neuronal function [88]. This line of evidence lets to hypothesize that the primary role of MICU3 is to enhance MCU opening to ensure mitochondrial Ca^2+^ uptake in response to both small and fast cytosolic Ca^2+^ rises, typical of synaptic neuronal stimulation (Figure 4).

### 3.7. EMRE

The Essential MCU REgulator (EMRE) is an additional constituent of the uniporter, discovered by quantitative mass spectrometry analysis of affinity-purified MCU complex components [91] (Figure 1). EMRE is a metazoan-specific protein of 10 kDa, ubiquitously expressed in all mammalian tissue, with one transmembrane domain, a mitochondrial targeting sequence, and a highly conserved *C*-terminus [91]. Moreover, it is required for the binding of MICU1 to MCU (Figure 2). Initial biochemical and cellular studies revealed that it is required for MCU function [91,92]. Indeed, in yeast cells, reconstituted with human MCU protein, the expression of MCU alone is not sufficient for uniporter activity, because the MCU channel is active only when also EMRE is co-expressed with the MCU pore-forming unit [92]. Interestingly, the knockdown of EMRE led to the loss of mitochondrial Ca^2+^ uptake to a similar extent to what was observed in *MCU*-silenced HEK-293T and HeLa cells [91]. The following studies tried to give a more detailed explanation of the EMRE function within the MCU complex [93]. EMRE was shown to control MCU activity by sensing Ca^2+^ elevation inside the matrix via its *C*-terminal domain and coordinating the other MCU regulators. Indeed, when EMRE acidic *C*-terminus was either deleted or substituted with neutral residues, mitochondrial Ca^2+^ permeation through the uniporter increased and the Ca^2+^ concentration inside the matrix consistently augmented [93]. After that, a dual-mode of action for the small MCU regulator has been proposed, in contrast with the previously described model [94]. According to this view, EMRE stimulates MCU channel activity via the interaction of transmembrane helices from both proteins, proposing a different EMRE orientation in which the *N*-terminus is present inside the matrix, while its *C*-terminal portion faces the IMS. In addition, EMRE was proposed to exert its MCU-regulation activity by binding MICU1 via its conserved *C*-terminal poly-aspartate tail (Figure 2). In the same year, another interesting aspect of EMRE-dependent MCU regulation has been revealed: the m-AAA protease-mediated degradation of EMRE is an essential event to guarantee the correct MCU-MICU proteins’ assembly [95]. Indeed, the deficiency of m-AAA leads to EMRE accumulation, which prevents the MCU-MICUs association by competing for MCU binding. This generates a constitutively active MCU-EMRE channel that finally induces mitochondrial Ca^2+^ overload and eventually death of neuronal cells.

Very recently, the cryo-EM structure of the human MCU complex added new insights into the interaction of EMRE within the complex and defined the structural elements by which EMRE exerts its action on the channel [53]. In particular, it confirmed that EMRE crosses the IMM with its *N*-terminus facing the mitochondrial matrix. Secondly, it proposed that EMRE interaction with MCU occurs along with three major contact points of EMRE *N*-terminus, thus allowing Ca^2+^ ions to exit the channel vestibule. Finally, EMRE seems also to be crucial for MCU dimerization since, in its absence, the MCU channel is found as a monomer [53].

These new findings added important notions on the mechanisms of action and regulation of the MCU complex and highlighted the need for a precise orchestration of the holocomplex assembly to ensure its fundamental role in regulating cellular Ca^2+^ signals, cell metabolism, and cell survival.

### 3.8. MCUR1

The mitochondrial calcium uniporter regulator 1 or MCUR1 has been firstly described as an IMM-resident protein of 35 kDa, encoded by the *CCDC90A* gene, resulted from an RNAi screening searching for mitochondrial membrane components involved in mitochondrial Ca^2+^ uptake regulation [96] (Figure 1).

Only recently, crystallographic analysis of human MCUR1 revealed the sites of its interaction with MCU. Human MCUR1 structure shows that this coiled-coil-containing protein contains different domains called head, neck, stalk, and a membrane anchor. The head segment is presented as the responsible portion of the direct binding to the *N*-terminus of MCU, while the length of the stalk portion seems to be of crucial importance for the interaction with the uniporter, even though it is not directly involved in MCU-MCUR1 connection [97]. Moreover, it has been shown that the MCUR1-dependent regulation of MCU depends on MCUR1 protein level and stability, similarly to what is reported for EMRE during MCU complex assembly [97].

Despite the precise function of MCUR1 remains to be clarified, several lines of evidence suggest that it regulates the mitochondrial Ca^2+^ entry through MCU [96,98]. This hypothesis has been strengthened by the fact that MCUR1 expression controls the Ca^2+^ threshold required for permeability transition via PTP, thus proposing a possible mechanism in which MCUR1 could bridge the MCU and PTP complexes [99]. In contrast to this view, Paupe and collaborators showed that MCUR1 is not a direct regulator of MCU, but rather a cytochrome c oxidase (COX) assembly factor [100]. This concept originated from the observation that mammalian and yeast cells (note that the latter lack MCU expression), in which the *CCDC90A* gene was silenced, showed an incorrect COX assembly leading to a decrease of the mitochondrial membrane potential and, consequently, to the collapse of the driving force for mitochondrial Ca^2+^ uptake [100].

The latest findings assigned back to MCUR1 a relevant role in the control of cell metabolism [101] and autophagy [96,99,102] through modulation of mitochondrial Ca^2+^. Intriguingly, the putative *Saccharomyces cerevisiae* MCUR1 homologs, Put6 and Put7, act as regulators of mitochondrial proline metabolism. Indeed, their loss results in a massive defect in yeast proline utilization, which is rescued by the heterologous expression of human MCUR1 [101]. In mammalian cells, MCUR1 downregulation has been also linked to AMPK phosphorylation and LC3 processing [96], which directly links MCUR1 levels to the activation of the autophagy pro-survival pathway, in particular under stress conditions, as reported in vascular endothelial cells during oxygen and glucose deprivation [102].

Interestingly, recent evidence of a possible implication of MCUR1 in human pathology has been provided in the hepatocellular carcinoma cell (HCC) model. In this context, MCUR1-dependent mitochondrial Ca^2+^ uptake has been reported to stimulate in vitro invasion and in vivo metastasis by promoting EMT via the ROS/Nrf2/Notch pathway and Snail transcription, pointing to MCUR1 as a potential therapeutic target for hepatocellular carcinoma treatment [103].

## 4. Mitochondrial Ca^2+^ Uptake in Physiology: Skeletal Muscle as a Paradigm

The fact that skeletal muscle comprises around 40% of human body mass [104], is primarily responsible for everyday locomotor activity and plays a pivotal role in the maintenance of whole-body health, temperature, and energy homeostasis, makes it a key model system for biomedical and life science investigations. Skeletal muscle is indeed composed of excitable cells, namely the myofibers, which react to the nerve electrical stimulation by contracting, thus generating muscle force. Myofibers are giant, multinucleated syncytial cells with paradigmatic morphology, from which they derived their name, and that contain a highly organized cytoplasmic architecture. The muscle fiber cytoplasm is filled with a repetitive and regularly aligned array of contracting units, the "sarcomeres", consisting of fibrillar and structural proteins, of which the myosin and actin filaments are the best known [105]. Myofibers are terminally differentiated cells that definitively exited the cell cycle, so the muscle is a post-mitotic tissue. Despite that, skeletal muscles can regenerate after damage and restore, in a relatively short time, both the size and the properties they had before the injury [106], thanks to the proliferation and differentiation of a population of resident muscle progenitor cells called satellite cells [107,108].

Of most relevance, muscles can contract to generate force, and, importantly, relax afterward to return to their resting length and condition. Obviously, contraction activity is a highly demanding process that massively consumes the cellular energy stored in the form of ATP molecules. Myosin, the major sarcomeric filament and the effective motor core of the myofiber, is the most abundant protein in muscle, comprising 25% of the total tissue protein [109] and it possesses the catalytic activity to hydrolyze ATP. The energy of ATP hydrolysis serves to promote actin filament sliding, thus producing sarcomere shortening and fiber contraction [110,111]. However, a consistent amount of ATP is also necessary for the activity of muscle fiber ion pumps (Na^+^/K^+^ ATPase and Ca^2+^ ATPase in the sarcolemma, sarcoplasmic reticulum Ca^2+^ ATPase in the ER). In addition, muscle is an extremely plastic tissue; muscle fibers can adapt and promptly respond to different workload demands shaping both their metabolic output and gene expression profile to match with changes in energy requirements of different types of exercise as well as during different phases of a single bout of exercise. For this reason, muscle metabolism needs to be highly efficient but flexible and rapidly adaptable. The main sources of ATP in muscles are the catabolism of glucose (primarily aerobic during the peak of exercise activity, but also anaerobic at the initial step of the exercise sprint) and the oxidation of fatty acids (especially in the recovery phase after exercise). Other metabolic routes participate in the overall tissue energetics to a much-reduced extent [112].

Finally, we will briefly describe the key molecular events leading to muscle contraction and muscle metabolic adaptation, with particular emphasis on the role played by Ca^2+^ signaling on these two processes. The start of muscle contraction occurs at the membrane of myofibers, where the motor neuron-released neurotransmitter triggers sarcolemma depolarization and the consequent opening of voltage-gated ion channels (the DHPR). DHPR are structurally and functionally coupled to the Ca^2+^ release channels of the endo-sarcoplasmic reticulum (SR), the Ryanodine receptor (RyR), so that RyR also opens [113]. This induces a rapid and massive release of Ca^2+^ from the SR with the subsequent intracellular Ca^2+^ elevation and activation of troponins which, in turn, allow the actomyosin sliding and promote sarcomere contraction. This process is commonly defined as excitation-contraction coupling or ECC [114]. Despite the role of Ca^2+^ in skeletal muscle is, in the first place, associated with actomyosin contraction and ECC, the cation exerts at least two other crucial functions in muscle physiology. On the one hand, the elevation of intracellular Ca^2+^ regulates a plethora of Ca^2+^-sensitive effectors, such as nuclear transcription factors, calmodulins, and kinases which sequentially activate gene expression and downstream signaling cascades [115]. Secondly, intracellular Ca^2+^ increase leads to mitochondrial Ca^2+^ uptake and the consequent stimulation of muscle aerobic metabolism and oxidative respiration [116,117,118]. Indeed, the cytosolic Ca^2+^ rise induced in the myofiber by nerve activity leads to Ca^2+^ binding to calmodulin and consequent activation of the Ca^2+^-calmodulin-dependent phosphatase Calcineurin. Calcineurin dephosphorylates its target, the Nuclear Factor of Activated T-cells or NFAT [119] that, once dephosphorylated, is allowed to translocate to the nucleus and modulate the transcription of target genes, such as those of myosin heavy chains [120,121]. This Ca^2+^-dependent, Calcineurin-NFAT-mediated regulation of gene transcription was shown to be crucial for the expression of muscle-specific structural genes that characterize the different fiber-type populations [120,121].

**Figure 5:**
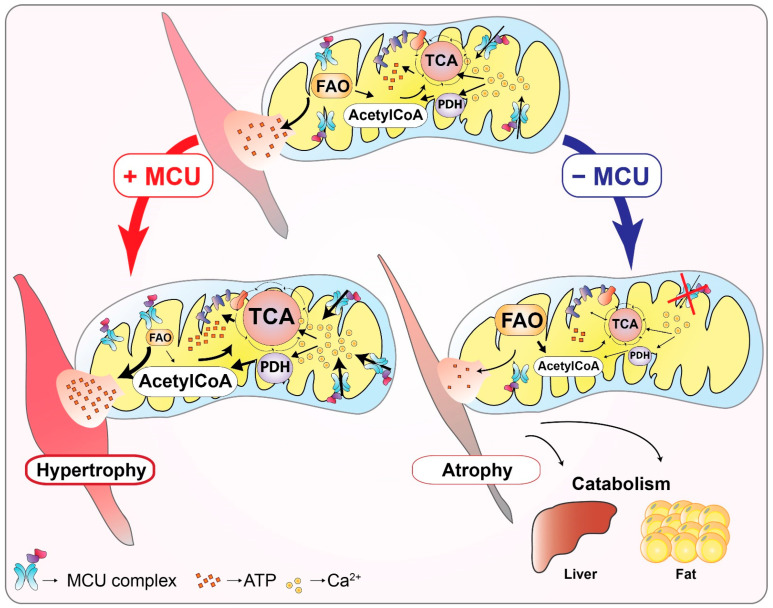
The role of MCU in the control of skeletal muscle trophism and metabolism. Schematic representation of the effects of manipulation of MCU activity on mitochondrial metabolism and trophism of the skeletal muscle tissue. In physiological conditions, Ca^2+^ entry into mitochondria stimulates glucose and fatty acids utilization to produce energy through the TCA cycle, via the activity of pyruvate dehydrogenase (PDH) and fatty acid oxidation (FAO), respectively. The enhanced mitochondrial Ca^2+^ uptake by MCU overexpression promotes muscle fiber oxidative metabolism leading to muscle hypertrophy via Akt pathway activation and upregulation of PGC1α4. On the contrary, the reduction of Ca^2+^ entry in the mitochondria by *MCU* deletion induces muscle metabolism rewiring towards an increased FAO, provokes muscle atrophy, impairs exercise performance, and causes global metabolic alterations, leading to an enhanced liver and adipose tissue catabolism.

On the other hand, the activity-dependent intracellular Ca^2+^ mobilization induces Ca^2+^ uptake by myofiber mitochondria and enhances mitochondrial metabolism through the TCA cycle and respiratory chain [116,117,118], as discussed before (Figure 5). Thus, the dynamics of intracellular Ca^2+^ and in particular of mitochondrial Ca^2+^ are necessarily associated with the regulation of skeletal muscle metabolism. This concept has been recently explored and we will report here some of the latest advances in the study of mitochondrial Ca^2+^ signaling and its influence on muscle metabolism and adaptation to both physiologic workload demands and pathologic conditions.

The molecular identification of the mitochondrial Ca^2+^ channel components [40,41,42,84,88,91,96,122] (see the previous paragraph for details) definitively boosted this field of research, allowing the genetic manipulation of mitochondrial Ca^2+^ uptake in vivo, including in skeletal muscle. Despite the first report of *MCU* gene deletion in the total knock out mouse model showed an unexpectedly mild phenotype, the MCU^−/−^ mice display a reduction of both resting mitochondrial Ca^2+^ concentration and mitochondrial Ca^2+^ uptake after stimulation at the cellular level, and increased serum lactate at the systemic level [55]. These data suggested the presence of impaired regulation of mitochondrial oxidative metabolism although the initial measurement of O_2_ consumption in MCU^−/−^ and wt MEF cells did not reveal significant differences [55]. Nevertheless, the presence of a defective mitochondrial energetic control was clearly demonstrated in the muscle tissue of MCU^−/−^ mice, by the presence of an elevated amount of the phosphorylated inactive form of PDH, which reduces TCA cycle activity, and by a reduced exercise performance and muscle force of the KO animals [55] (Figure 5).

In addition, the fact that the MCU^−/−^ mouse model presents a highly variable phenotypic severity that is dependent on the genetic background, i.e., relatively mild in the mixed background mice [55] but embryonic lethal in the pure C57BL/6 mice [67], brought back up the concern about the possibility that long-term genetic manipulation of MCU may induce unpredictable developmental compensatory mechanisms that might mask the physiologic effects of the targeted gene deletion.

A few years later, the modulation of mitochondrial Ca^2+^ uptake in a skeletal muscle-specific manner has been made possible by the selective manipulation of MCU expression in muscle fibers. The in vivo application of this approach was instrumental to decipher the complex role of mitochondrial Ca^2+^ signaling in controlling muscle tissue homeostasis as well as in rewiring the systemic metabolism of the whole organism [61,62] and proved that variations in mitochondrial Ca^2+^ dynamics directly control muscle trophism [117]. Indeed, the transient downregulation of mitochondrial Ca^2+^ uptake by AAV-mediated *MCU* silencing in mouse muscle induced remarkable fiber atrophy and impaired activation of the pyruvate dehydrogenase complex [117], in agreement with what was reported for the MCU^−/−^ mice [55]. On the contrary, AAV-induced MCU overexpression produced significant muscle hypertrophy, which was shown to be mediated by the IGF1-Akt pathway and by the transcriptional activation of one of the key regulators of exercise-induced muscle hypertrophy [123], the peroxisome proliferator-activated receptor-gamma coactivator 1 alpha 4 gene (PGC1α4) [117] (Figure 5).

In line with the high relevance of the MCU complex activity in the maintenance of muscle function demonstrated in animal and in vitro experimental models, *MICU1* was identified as the disease-causing gene of a human disease phenotype characterized by proximal myopathy, learning difficulties, and a progressive extrapyramidal movement disorder [78]. From a clinical point of view, the pathology has an early onset, characterized by proximal muscle weakness, elevated serum creatine kinases, and intellectual impairment, progressing with extrapyramidal motor involvement and involuntary movements causing severe disability [78]. The whole exome-sequencing of affected patients revealed different loss-of-function mutations in the *MICU1* gene. Myopathic features such as the presence of centrally nucleated fibers, areas devoid of mitochondria (cores), and significant variability in myofiber size are found in the muscles of the analyzed patients. Despite that, the overall fiber-type distribution is maintained. However, the characterization of dermal fibroblast cells from *MICU1*-deficient patients together with that of muscle fibers from skeletal muscle-specific MICU1 KO mouse model (skmMicu1^−/−^) revealed a complex cellular mechanism underlying skeletal muscle dysfunction in the absence of this crucial regulator of mitochondrial Ca^2+^ entry [124]. The analysis of the mitochondrial Ca^2+^ dynamics of patient fibroblasts and skmMicu1^−/−^ mouse myofibers showed the loss of both MCU gatekeeper activity and cooperative MCU activation [124]. Indeed, MICU1-deficient cells showed an increased mitochondrial Ca^2+^ resting level and an enhanced rate of mitochondrial Ca^2+^ uptake [78]. Nevertheless, the values of mitochondrial Ca^2+^ peak in stimulated MICU1 KO cells are not different from those of wt cells [78]. In addition, the cytosolic Ca^2+^ transients after cell stimulation are reduced in *MICU1*-deficient cells as a consequence of the enhanced mitochondrial buffering capacity [78]. The cellular and global metabolism seem not to be affected by *MICU1* deletion, as shown by an O_2_ consumption rate of patient cells [78] and a blood lactate level of skmMicu1^−/−^ mice [124] similar to controls. Despite that, the skmMicu1^−/−^ mice showed enhanced lactate and creatine kinase levels, accompanied by reduced running time and overall decreased performance when assessed after treadmill running until exhaustion. The mechanism underlying muscle failure was unveiled by Evan’s blue dye analysis of muscle fiber integrity, which revealed clear signs of sarcolemma damage in skmMicu1^−/−^ fibers, not found in wt fibers [124]. MICU1 appears then as a crucial mediator of the role of mitochondrial Ca^2+^ in myofiber plasma membrane recovery after exercise-induced damage in in vivo mouse skeletal muscle, but also in human fibroblasts of patients with MICU1 mutation after laser-induced injury [124]. These findings are in line with the notion that impaired mitochondrial Ca^2+^ signaling causes defects in the plasma membrane repair machinery in the C2C12 muscle cell model. Interestingly, the mitochondrial Ca^2+^-mediated healing of membrane damage has been shown to rely mainly on the activity of the small GTPase RhoA and the dynamics of actin filaments, while it appears substantially independent from the mitochondrial ATP and energy production [125]. The contribution of mitochondrial Ca^2+^ signaling to the repair of fiber sarcolemma brings an additional level of complexity to the regulation of muscle fiber homeostasis by mitochondrial Ca^2+^ providing new opportunities for future investigations and, possibly, offering new targetable pathways for therapeutic interventions against neuromuscular disorders.

## 5. Conclusions and Future Perspectives

In this review, we provided a historical overview of the achievements in the study of mitochondrial Ca^2+^ signaling. We presented a synopsis of the discoveries that, in the last decade, led to the identification of most of the components that constitute the mitochondrial Ca^2+^ uptake machinery (see Figure 1), highlighting the regulatory factors that govern the fine-tuning of Ca^2+^ entry in the organelle (see Figure 2, Figure 3 and Figure 4) and, finally, focusing on the relevance of mitochondrial Ca^2+^ in the context of skeletal muscle. We described the recent advances in the comprehension of Ca^2+^ signaling the control of muscle metabolism at the level of myofiber, tissue, and whole organism (see Figure 5).

Despite the increasing knowledge on mitochondrial Ca^2+^ signaling in cell and organ physiology, several questions on the function and regulation of mitochondrial Ca^2+^ are still unanswered.

One of the key unsolved issues still puzzling the scientific community concerns the variability of the germline MCU KO mouse model phenotype, which depends on the genetic background of the animals. Although a definitive explanation could not be provided so far, we might speculate on the compensatory mechanisms responsible for the relatively mild phenotype of the outbred MCU-deficient mice. One of those envisages the possibility that, in the absence of MCU, its alternative MCUb isoform may exert a limited Ca^2+^ channel activity. Indeed, although MCUb has been demonstrated to act as a dominant-negative subunit of the uniporter both in cellular and in vitro systems [69], it remains to experimentally validate whether the protein retains some Ca^2+^ permeability, which might result in a low Ca^2+^ flux rate, below the detection of the electrophysiological measures used to assess MCUb channel activity, but sufficient to allow some Ca^2+^ entry into the matrix in the absence of the canonical MCU.

A second, more general possible compensatory mechanism consists in the adaptation of embryonic cells to the lack of MCU activating alternative strategies and molecular routes to maintain the homeostatic matrix Ca^2+^ level, such as the modulation of IMM ion transporters and exchangers expression (as was shown for the Na^+^/Ca^2+^ exchanger NCLX [56].

However, the observed lethality of inbred MCU KO mice leaves the question about MCU function during development still open. This is an issue that definitively deserves to be more deeply investigated.

Another aspect of the biology of mitochondrial Ca^2+^ that remains to be clarified despite being relevant for many developmental processes concerns its specific role in cell death. The fact that intracellular Ca^2+^ signaling plays a key role in all types of cell death (apoptosis, necrosis, necroptosis, and autophagy-mediated) [15] is a widely accepted concept. However, whether and to what extent mitochondria Ca^2+^ uptake dictates the cell decision either to survive or to perish is still debated. Studies characterizing the heart of MCU-constitutive KO mice [55] and cardiac-specific *MCU*-deleted animals [56,57] showed contrasting results about the role of MCU in cardiomyocyte cell death following in vivo cardiac ischemia-reperfusion injury. Indeed, the hearts of germline MCU KO mice resulted not protected from the ischemia-reperfusion damage despite they display resistance to elevated Ca^2+^-mediated activation of the mitochondrial Permeability Transition Pore (mtPTP) [55]. On the contrary, the targeted ablation of MCU in adult cardiomyocytes showed the inhibition of Ca^2+^-induced mtPTP opening together with a reduced cardiomyocyte death after ischemia-reperfusion [56,57].

The different results obtained from constitutive versus inducible tissue-specific MCU KO mouse models suggest the possibility that the MCU deletion may lead to long-term changes in the gene expression program. The characterization of these changes and the mechanisms by which they take place are yet to be defined.

Finally, although the quantitative proteomic data provided by the group of Mootha clearly indicate that all the components of the MCU holocomplex (uniplex) and associated regulators have been already identified [91], the possibility that additional still unknown modulators and tissue-specific factors may control the uniporter channel activity still exists. In addition to that, the eventuality of cell-type-specific alternative splicing isoforms of known uniplex proteins (as in the case of MICU1-MICU1.1 in skeletal muscle fibers [83]) should be considered in the plethora of the MCU regulatory mechanisms still to be characterized.

These considerations support the concept that the mitochondria of different cell types might have different Ca^2+^ levels and Ca^2+^ responses to match their metabolic requirement and physiological function. Indeed, timely and tissue-specific regulation of the mitochondrial Ca^2+^ uniporter activity is ensured by the different proportions of the MCU holocomplex components, as depicted in Figure 4, thus warranting the appropriate metabolic activation for each cell type.

Concluding, our understanding of mitochondrial Ca^2+^ role and regulation has grown massively in the last ten days; however, additional investigations are still needed to unravel the unresolved issues. Hopefully, scientists will take up this challenge and their work will provide further knowledge on the signaling and regulation of mitochondrial Ca^2+^ to better understand its role in cells and organs in physiology as well as to possibly develop new routes of intervention in pathological conditions.

## Data Availability

Not applicable.
